# Plasmacytoid dendritic cells orchestrate innate and adaptive anti-tumor immunity induced by oncolytic coxsackievirus A21

**DOI:** 10.1186/s40425-019-0632-y

**Published:** 2019-07-01

**Authors:** Louise M. E. Müller, Matthew Holmes, Joanne L. Michael, Gina B. Scott, Emma J. West, Karen J. Scott, Christopher Parrish, Kathryn Hall, Sina Stäble, Victoria A. Jennings, Matthew Cullen, Stewart McConnell, Catherine Langton, Emma L. Tidswell, Darren Shafren, Adel Samson, Kevin J. Harrington, Hardev Pandha, Christy Ralph, Richard J. Kelly, Gordon Cook, Alan A. Melcher, Fiona Errington-Mais

**Affiliations:** 1 Section of Infection and Immunity, Leeds Institute of Medical Research (LIMR), University of Leeds, St. James’s University Hospital, Level 5, Wellcome Trust Brenner Building (WTBB), Leeds, LS9 7TF UK; 2 Haematological Malignancy Diagnostics Service, St. James’s University Hospital, Leeds, UK; 3 Department of Haematology, St. James’s University Hospital, Leeds, UK; 4 School of Biomedical Science and Pharmacy, University of Newcastle, Newcastle, Australia; 5 Translational Immunotherapy Team, The Institute of Cancer Research and Royal Marsden Hospital/Institute of Cancer Research NIHR Biomedical Research Centre, London, UK; 6 Surrey Cancer Research Institute, Leggett Building, Faculty of Health and Medical Sciences, University of Surrey, Guildford, UK; 7 Section of Experimental Haematology, LIMR, University of Leeds, St. James’s University Hospital, Leeds, UK

**Keywords:** Oncolytic viruses, Coxsackievirus A21, Plasmacytoid DC, Innate immunity, Adaptive immunity

## Abstract

**Background:**

The oncolytic virus, coxsackievirus A21 (CVA21), has shown promise as a single agent in several clinical trials and is now being tested in combination with immune checkpoint blockade. Combination therapies offer the best chance of disease control; however, the design of successful combination strategies requires a deeper understanding of the mechanisms underpinning CVA21 efficacy, in particular, the role of CVA21 anti-tumor immunity. Therefore, this study aimed to examine the ability of CVA21 to induce human anti-tumor immunity, and identify the cellular mechanism responsible.

**Methods:**

This study utilized peripheral blood mononuclear cells from i) healthy donors, ii) Acute Myeloid Leukemia (AML) patients, and iii) patients taking part in the STORM clinical trial, who received intravenous CVA21; patients receiving intravenous CVA21 were consented separately in accordance with local institutional ethics review and approval. Collectively, these blood samples were used to characterize the development of innate and adaptive anti-tumor immune responses following CVA21 treatment.

**Results:**

An Initial characterization of peripheral blood mononuclear cells, collected from cancer patients following intravenous infusion of CVA21, confirmed that CVA21 activated immune effector cells in patients. Next, using hematological disease models which were sensitive (Multiple Myeloma; MM) or resistant (AML) to CVA21-direct oncolysis, we demonstrated that CVA21 stimulated potent anti-tumor immune responses, including: 1) cytokine-mediated bystander killing; 2) enhanced natural killer cell-mediated cellular cytotoxicity; and 3) priming of tumor-specific cytotoxic T lymphocytes, with specificity towards known tumor-associated antigens. Importantly, immune-mediated killing of both MM and AML, despite AML cells being resistant to CVA21-direct oncolysis, was observed. Upon further examination of the cellular mechanisms responsible for CVA21-induced anti-tumor immunity we have identified the importance of type I IFN for NK cell activation, and demonstrated that both ICAM-1 and plasmacytoid dendritic cells were key mediators of this response.

**Conclusion:**

This work supports the development of CVA21 as an immunotherapeutic agent for the treatment of both AML and MM. Additionally, the data presented provides an important insight into the mechanisms of CVA21-mediated immunotherapy to aid the development of clinical biomarkers to predict response and rationalize future drug combinations.

**Electronic supplementary material:**

The online version of this article (10.1186/s40425-019-0632-y) contains supplementary material, which is available to authorized users.

## Background

Oncolytic virotherapy (OVT) has made significant progress for the treatment of solid malignancies over recent years, not least following the FDA approval of a genetically engineered herpes simplex virus (Imlygic®) for the treatment of melanoma [1]. Moreover, a recent clinical report demonstrating that OVT can improve the efficacy of the anti-PD-1 immune checkpoint antibody, pembrolizumab, increases their potential clinical applicability [2]. Oncolytic viruses (OVs) can utilize two distinct mechanisms to induce their anti-tumor effects, namely: 1) direct cytotoxicity (oncolysis) following replication and lytic killing of tumor cells [3–5], and 2) induction of anti-tumor immunity, which can be mediated by innate and adaptive immune mechanisms [6–8]. Specifically, OVs alter the tumor microenvironment through the induction of pro-inflammatory cytokines and a reduction in immunosuppressive factors, such as VEGF and IL-10. Furthermore, OVs enhance natural killer (NK) cell-mediated tumor killing [9–12] and stimulate the generation of cytotoxic T lymphocytes (CTLs) through the release of tumor-associated antigens (TAAs), with OVs providing the immunological “danger” signal [8, 12–14]. TAAs are subsequently processed by antigen-presenting cells (APC) and presented to CD4^+^ T cells, or cross-presented to CD8^+^ T cells, to promote an adaptive immune response, including immunological memory, against the tumor [15, 16]. Despite numerous efforts to enhance the direct lytic potential of OVT, including suppression of host immune responses, the induction of anti-tumor immunity has emerged as a pivotal mechanism for long-term clinical efficacy [17].

The *Kuykendall* strain of coxsackievirus A21 (CVA21) was manufactured to clinical grade (CAVATAK®) by Viralytics Ltd. and was recently acquired by Merck/MSD. CVA21 has been tested in a number of clinical trials [18–20] and, like Imlygic®, CAVATAK® has yielded promising results in the treatment of melanoma and other solid tumors [21, 22]; a Phase I clinical trial (STORM; Systemic Treatment Of Resistant Metastatic disease) of intravenously administered CAVATAK® in combination with pembrolizumab is ongoing [18]. CVA21 may cause mild upper respiratory tract infection in immunocompetent adults and approximately 15% of the population possess pre-existing antibodies against CVA21 [22, 23]. CVA21, an enterovirus belonging to the family *Picornaviridae*, has a positive-sense single-stranded RNA genome and utilizes Decay Accelerating Factor (DAF) to bind to host cells and Intercellular Adhesion Molecule 1 (ICAM-1) for internalization. Thus, overexpression of ICAM-1, observed on many malignantly transformed cells, can serve as a predictor of tumor cell susceptibility to CVA21-mediated oncolysis [3, 24, 25]. However, the inability of CVA21 to infect mouse cells has limited testing of its immunotherapeutic potential in immune-competent pre-clinical animal models.

Currently, OVT remains relatively under-investigated in the context of hematological malignancies with the majority of studies focusing on their direct lytic potential [26–29]. As such, the role for OV-induced immunotherapy, in potentially immunocompromised patients, is less clear. To date, preclinical studies have evaluated a small selection of OVs for the treatment of AML, including myxoma virus [26], vesicular stomatitis virus (VSV) [30], reovirus [6] and herpes simplex virus-1 (HSV-1) [31]; however, disappointingly, only one OV, VSV genetically modified to express IFN-β and sodium iodide symporter genes, has progressed to early clinical trials [32]. By contrast, the role for OVs in the treatment of multiple myeloma (MM) is more established with multiple clinical trials ongoing or completed [33]. Both AML and MM are in need of novel therapeutic interventions which are safe and well-tolerated, such as OVT [34, 35]. Therefore, we examined the immunotherapeutic potential of CVA21 against AML, which was relatively non-permissive to CVA21 infection and oncolysis, and MM, which was sensitive to CVA21-induced oncolysis. This study provides pivotal insight into: 1) the potential for CVA21-induced anti-tumor immunity against hematological malignancies; 2) the immunotherapeutic properties of CVA21; and 3) the cellular requirements for CVA21-induced anti-tumor immunity.

## Methods

### Cell culture and patient samples

All cell lines were grown according to ATCC/ECACC recommendations and ICAM-1-expressing KG-1 cells (ICAM-1/KG-1) were generated using lentiviral transduction.

Peripheral blood mononuclear cells (PBMC), obtained from healthy volunteers or leukocyte apheresis cones (National Health Service Blood and Transplant), were isolated using Lymphoprep™ (Fresenius-Kabi AS, Halden, Norway) density-gradient centrifugation as previously described [7]. PBMC were used at 2 × 10^6^ cells/mL and PBMC-conditioned medium (CM), collected 48 h after CVA21 treatment, was stored at − 20 °C prior to use. For type I IFN neutralisation, PBMC were pre-incubated for 30 mins with polyclonal antibodies prior to addition of CVA21 for 24 h [10]. For ICAM-1 blockade, PBMCs were either treated with 10 μg/mL LEAF™ purified anti-human ICAM-1-blocking antibody, a mouse IgG1-isotype control antibody (both BioLegend), or left untreated for 30 min at 37 °C. Subsequently, the PBMC were exposed to CVA21 for 24 h, before evaluation of NK cell activation and cytokine secretion.

Blood samples were obtained from patients diagnosed with AML or patients taking part in the STORM Phase 1 clinical trial (NCT02043665/Keynote-200/VLA009A) following additional informed consent. Written, informed consent was obtained from all patients in accordance with local institutional ethics review and approval (06/Q1206/106). Peripheral blood samples collected from patients recruited into the STORM clinical trial were collected from Cohort 1 and 3 (Cycle 1), who received 1 × 10^8^ or 1 × 10^9^ TCID_50_ clinical grade CVA21, respectively. Samples were collected prior to the first CVA21 infusion in Cycle 1, then at 1 h, 3 days and 22 days after the first infusion; samples collected on day 3 and 22 were taken before scheduled CVA21 treatments.

CD14^+^ monocytes and plasmacytoid dendritic cells (pDC, Lin^−^, BDCA-2^+^, BDCA-4^+^, CD123^+^, CD4^+^, CD45RA^+^, BDCA-3^dim^, BDCA-1^−^, CD2^−^) were isolated or depleted from whole PBMC using magnetic cell sorting on MACS® columns (Miltenyi Biotec, Bergish Gladbach, Germany), according to the manufacturer’s instructions. PBMC (±CD14^+^ monocytes or pDC) were used for collection of CM, assessment of NK cell activation and for CTL priming assays (see below).

### Gene expression analysis

mRNA was isolated using the RNeasy Plus kit (Qiagen, Hilden, Germany) and converted to cDNA using the SuperScript™ III Reverse Transcriptase kit and oligoDT priming (Thermo Fisher Scientific, Waltham, MA). Gene expression was evaluated by qPCR using TaqMan™ reagents and the QuantStudio 5 Real-Time PCR System (Thermo Fisher Scientific, Waltham, MA).

### Coxsackievirus A21

Wild-type coxsackievirus A21, *Kuykendall* strain, was provided by Viralytics Ltd. (Sydney, Australia) or propagated in-house from wild-type CVA21 obtained from ATCC® (ATCC®VR-850™). For propagation, supernatants were harvested following CVA21 infection of Mel624 cells for 24 h. CVA21 was pelleted by centrifugation at 36000 rpm for 2 h (SW45 rotor, Optima™ L-80 ultra-centrifuge, Beckman Coulter) and harvested virus was purified using OptiPrep™ density gradient centrifugation, 35–15% gradient (36,000 rpm, 1.5 h, SW41 Ti rotor). Viral titer was determined using a standard plaque assay on Mel624 cells.

### MTS assay

MTS assays were performed according to the manufacturer’s protocol (Abcam, Cambridge, UK). Optical density was measured at 450 nm using a Multiscan EX microplate reader (Thermo Fisher Scientific).

### Cytokine detection

IFN-α secretion was detected using matched paired antibodies (MabTech AB) and standard ELISA techniques. PBMC-CM was also analyzed using multiplex bead arrays (Bio-Plex Pro™ Human Cytokine 27-plex and 23-plex Assay; Bio-Rad) as per the manufacturer’s instructions. Plates were analyzed using a Bio-Plex 100 reader with Bio-Plex Manager software.

### Priming of AML-specific cytotoxic T cells

Immature DC (iDC) generation and CTL priming assays were performed as described by Prestwich et al [8]. Briefly, tumor cells (±0.1 pfu/cell CVA21 for 24 h) were loaded onto CD14^+^ monocyte-derived iDC and co-cultured with autologous PBMC for 1 week. CTL were then re-stimulated with tumor-loaded iDC (±CVA21) and cultured for a further 7 days. Primed CTL were then harvested for ^51^Cr release assay, CD107 degranulation or peptide recall assays.

Where indicated, CTL generation was also performed in the absence of iDC, using only CVA21-treated tumor cells. To do this, CVA21-infected tumor cells, treated with 0.1 pfu/cell CVA21 for 24 h, were centrifuged to remove free virus, and incubated for a further 48 h prior to addition of PBMC. PBMC were cultured for 1 week and re-stimulated with tumor cells (±CVA21 treatment – as above).

For TAA peptide stimulation, autologous CD14^+^ cells were thawed from frozen, washed with medium, and allowed to rest for 60 min prior to incubation with 6 nmol/mL PepTivator® peptide pools (15-mer peptide sequences with 11 amino acids overlap, Miltenyi Biotec) for 60 min at 37 °C; PepTivator® Peptide Pools were reconstituted in dH_2_O at 30 nmol/mL and aliquots were stored at − 80 °C. Autologous CD14^+^ cells with or without PepTivator® peptide loading were then co-cultured with CTL and intracellular IFN-γ production was determined by flow cytometry.

### Flow cytometry analysis

For all flow cytometry analysis, cells were stained with relevant antibodies (see Additional file 1: Table S1 and analysis was performed on an Attune® Acoustic Focusing Cytometer (Thermo Fisher Scientific) or CytoFLEX S (Beckman Coulter). Cell viability was evaluated using a LIVE/DEAD® Fixable Dead Cell Stain Kit (Thermo Fisher Scientific). For NK cell and CTL CD107 degranulation assays, PBMC were co-cultured with target cell lines (2:1 ratio) for 1 h. Following this, Brefeldin A (1:1000, BioLegend, San Diego, CA) and relevant antibodies (see Additional file 1: Table S1) were added to each sample for 4 h. For intracellular IFN-γ staining, CTLs were co-cultured with target cells (2:1 ratio) or autologous CD14^+^ cells (±PRAME, Mucin-1 and MAGE-A1 peptide pools) for 1 h at 37 °C before addition of Brefeldin A (1:1000, BioLegend) and relevant antibodies (Additional file 1: Table S1) for a further 4 h at 37 °C. CTLs were then fixed in 1% paraformaldehyde (PFA) overnight and permeabilized using 0.3% saponin (Sigma-Aldrich) prior to intra-cellular IFNγ staining and acquisition by flow cytometry.

### ^51^Cr release assay

Target cells were labelled with 100 μCi ^51^Cr (PerkinElmer, Waltham, MA) then co-cultured with effector cells as previously described [6]; unlabeled K562 and Daudi cells were included in the analysis of CTLs to reduce non-specific killing. For NK cell ^51^Cr release assays, the data shown represents the effector:target ratio of 50:1 for AML (0.1pfu/cell CVA21), and 25:1 for MM (1pfu/cell). ^51^Cr was measured using a Microbeta^2^ scintillation counter (PerkinElmer) and the percentage cell lysis was calculated (cpm: counts per minute):

$$ \% lysis=100\times \frac{sample\  cpm- spontaneous\  cpm}{maximum\  cpm- spontaneous\  cpm} $$

### Statistical analysis

Statistical analysis was performed using Graphpad Prism 7.0 software. *p*-values were calculated using either Student’s *t*-test, one-way analysis of variance (ANOVA) or two-way ANOVA. Results were considered significant if *p* < 0.05 (* = *p* < 0.05, ** = *p* < 0.01, *** = *p* < 0.001, **** = *p* < 0.0001). Pearson’s *r* was calculated to evaluate correlations.

## Results

### Activation of immune effector cells in vivo following intravenous (i.v.) administration of CVA21

**Fig. 1:**
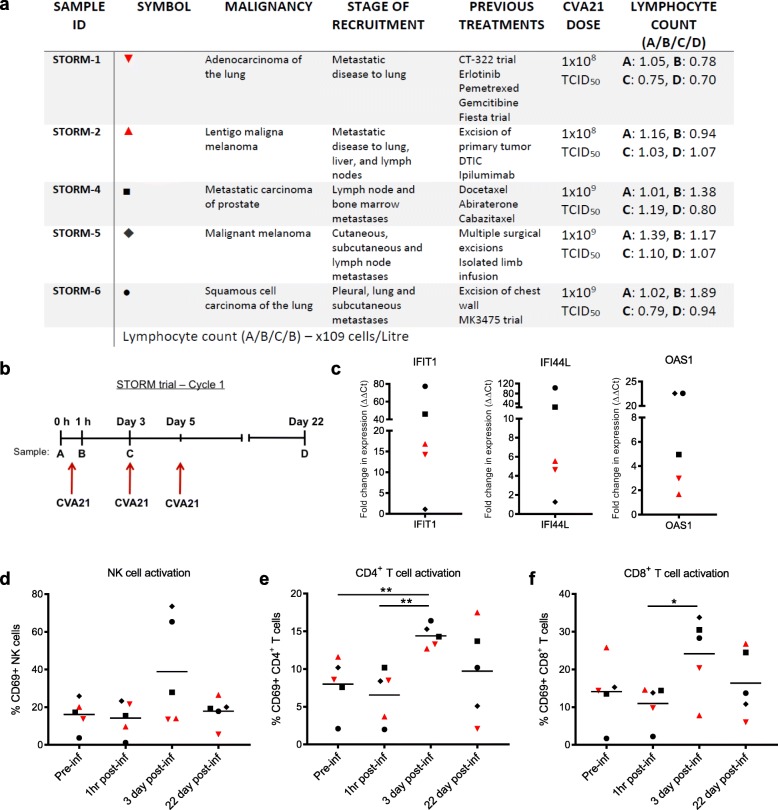
Intravenous CVA21 induces a type I IFN response and activates immune effector cells in vivo. **a**. As part of the STORM clinical trial, patients (*n* = 5) with advanced malignancies were administered with 1 × 10^8^ (red symbols) or 1 × 10^9^ TCID_50_ (black symbols) clinical grade CVA21 i.v. **b**. CVA21 was infused on day 1, 3 and 5 and blood samples were taken pre-infusion (**a**), 1 h (**b**), 3 days (**c**) and 22 days (**d**) after the first infusion. **c**. cDNA was made from PBMC collected pre-infusion (**a**) and on day 3 (**c**) and the expression of *IFIT1, IFI44L* and *OAS1* was measured by qPCR. Results were normalized to *18S* RNA expression and the fold increase in expression (calculated as ΔΔCt) compared to pre-infusion is presented. **d-f** CD69 expression on NK cells (**d**), CD4^+^ T cells (**e**) and CD8^+^ T cells (**f**) was analyzed at each time point. *denotes statistical significance

As CVA21 is dependent on human ICAM-1 for cell entry, only human model systems or immunocompromised human xenograft murine models allow in vivo testing of CVA21, with the latter preventing exploration of the role of adaptive anti-tumor immunity. Therefore, to initially confirm the immunotherapeutic potential of CVA21 in vivo, and in potentially immunosuppressed cancer patients [36–39], we took advantage of access to samples from STORM (VLA009A) clinical trial patients. This Phase I dose escalation study examined the safety of i.v. delivery of CVA21 to patients with mixed types of advanced cancers including melanoma, prostate and squamous cell carcinoma of the lung (Fig. 1a). STORM patients that consented to have additional research blood samples taken for analysis received 1 × 10^8^ (Cohort 1) or 1 × 10^9^ TCID_50_ (Cohort 3) clinical grade CVA21 on days 1, 3 and 5 (Cycle) and blood samples were collected prior to the first CVA21 infusion, then at 1 h, 3 days and 22 days after the first infusion (Fig. 1b). No consistent increase in IFN-α secretion, a key mediator of host anti-viral and anti-tumor responses, was observed in the patient plasma samples at these time points (data not shown). However, using qPCR, we observed an increase in the expression of interferon-stimulated genes (ISG), *IFIT1, IFI44L* and *OAS1*, 3 days after the first CVA21 infusion, compared with the baseline pre-infusion samples (Fig. 1c); this was more pronounced in patients who received a higher dose of virus and suggested the onset of a type I IFN response in the peripheral blood of CVA21-treated patients.

To analyze the effect of CVA21 on immune effector cells, the expression of CD69 (an early marker of lymphocyte activation) on NK cells, CD4^+^ T cells and CD8^+^ T cells was examined at each time point; a peak in immune cell activation was identified on day 3 (Fig. 1d-f). Stronger activation, particularly of NK cells (Fig. 1d), was observed in Cohort 3 where a higher dose of CVA21 was administered. Encouragingly, these data demonstrate that i.v. administration of CVA21 can induce a type I IFN response and activate immune cells in the peripheral blood of cancer patients. Importantly, data presented in Additional file 2: Figure S1 also demonstrate that CVA21 treatment is not toxic to healthy donor (HD) PBMC, and that HD-PBMC do not support CVA21 viral replication, in vitro.

Having confirmed that CVA21 could stimulate immune activation in patients, we wanted to further characterize the immunomodulatory properties of CVA21, and establish the role of CVA21-induced immunotherapy. To do this we first sought to identify models of disease which were sensitive or resistant to CVA21-induced direct oncolysis for use in established immunological assays. We identified AML cell lines, which expressed low levels of ICAM-1, as relatively insensitive to CVA21-induced direct oncolysis (see Additional file 3: Figure S2A and B, respectively), and MM cell lines (except OPM2), which expressed higher levels of ICAM-1, as highly susceptible to CVA21 oncolysis (see Additional file 3: Figure S2C and D, respectively).

### Pro-inflammatory cytokines induce bystander killing of CVA21-resistant cells

**Fig. 2:**
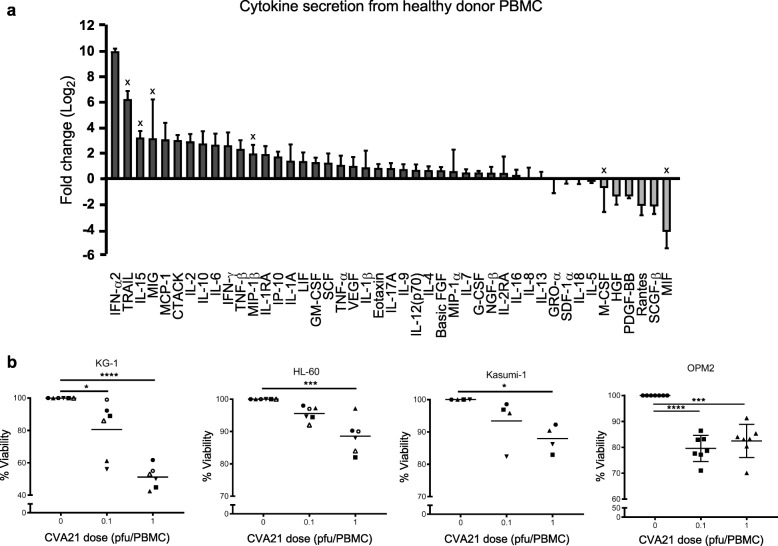
CVA21 treatment induces cytokine-mediated bystander killing of CVA21-resistant cells. **a**. Conditioned-media (CM) was collected from healthy donor PBMC after 48 h (±CVA21 treatment; 1 pfu/PBMC) and analyzed for cytokines using a 48-plex multiplex assay. The mean fold change (*n* = 3) compared to untreated PBMCs is shown. Sample readings outside of the detection range were estimated using the assay range limits and are indicated by x. **b**. CVA21-resistant cell lines were cultured for 96 h in PBMC-CM (0, 0.1 and 1 pfu/cell CVA21 treatment for 48 h; *n* = 6 for KG-1, HL-60 and OPM2, n = 5 for kasumi-1) and cell viability was assessed using an MTS assay. Error bars indicate SEM. *denotes statistical significance

The induction of ISGs following i.v. infusion of CVA21 implies an interferon response, and IFN-α2 has known cytotoxic potential against both AML and MM, with multiple IFN-α-based clinical trials having been completed [40, 41]. To date, the inflammatory milieu induced by CVA21 has not been thoroughly explored; moreover, its potential role in CVA21 efficacy remains unknown. To initially investigate this, we used a multiplex assay to examine the range of cytokines and chemokines induced from HD-PBMC following CVA21 treatment. These data confirmed that CVA21 elicited a strong cytokine response (Fig. 2a), with a number of potentially cytotoxic cytokines, including IFN-α2, TRAIL and IFN-γ, being identified. Having previously identified KG-1, HL-60, kasumi-1 and OPM2 cells as resistant to CVA21-direct oncolysis (Additional file 3: Figure S2B and D) these cells were subsequently used to examine the cytotoxic potential of CVA21-induced inflammation. KG-1, HL-60, kasumi-1 and OPM2 cells were cultured in PBMC-CM (±CVA21 treatment) for 96 h and cell viability was examined; CVA21-treated PBMC-CM significantly reduced the viability of all CVA21-resistant cell lines (Fig. 2b), which, given resistance to CVA21-direct oncolysis, was highly suggestive of CVA21-induced bystander cytokine killing. In support of this, reconstitution of culture media with recombinant IFN-α or IFN-γ (cytokines secreted in response to CVA21 treatment) also demonstrated a small, but significant, increase in killing of CVA21-resistant KG-1 cells (Additional file 4: Figure S3A).

Conversely, an alternative explanation for the death induced by PBMC-CM was a possible up-regulation of ICAM-1, which could ultimately confer susceptibility to CVA21-direct oncolysis. Indeed, we did observe a modest increase in ICAM-1 expression on cells treated with CVA21-treated PBMC-CM (up to 4-fold – data not shown). Therefore, to explore this possibility we used TNF-α, a known up-regulator of ICAM-1, and re-examined CVA21-direct oncolysis and assessed viral replication following TNF-α treatment. These results demonstrated that both THP-1 and Kasumi-1 cells remained resistant to CVA21-direct oncolysis despite a greater than 10-fold increase in ICAM-1 expression (Additional file 4: Figure S3B and C); moreover, plaque assays confirmed that there was no increase in CVA21 titre, compared to input virus, 72 h post-infection (data not shown). In addition, we also examined viral replication (by plaque assay) in CVA21-resistant THP-1 and Kasumi-1 cells following treatment with PBMC-CM (to increase ICAM- expression); similarly, no increase in viral titre was observed 72 h post infection (data not shown).

By contrast, KG-1 cells became susceptible to CVA21-direct oncolysis in accordance with increased ICAM-1 expression (Additional file 4: Figure S3D and E); suggesting that death of KG-1 cells (Fig. 2b) could, in part, be mediated by increased ICAM-1 expression following treatment with CVA21 treated PBMC-CM, which incorporates a range of inflammatory cytokines, including TNF-α. Interestingly, complimentary studies carried out using ICAM-1 transduced KG-1 cells (ICAM-1/KG-1), to remove possible off-target effects of TNF-α, examined CVA21 susceptibility in the presence of anti-viral type I IFN-α2 (at levels comparable to those identified in CVA21-treated PBMC-CM); these studies demonstrated a small increase in cytotoxicity following IFN-α treatment alone (as observed in Additional File 4: Figure S3A) but a significant abrogation of CVA21-direct oncolysis (Additional file 4: Figure S3F). Therefore, overall we believe that the inherent resistance of some cell lines to CVA21 (despite high levels of ICAM-1), the abrogation of CVA21-direct oncolysis by type I IFN-α (present in CVA21-treated PBMC-CM), and the direct cytotoxic potential of CVA21-induced cytokines, are more suggestive of bystander cytokine killing and indicate that the inflammatory changes stimulated by CVA21 treatment could contribute to CVA21-induced immunotherapy.

### CVA21-mediated activation of NK cells and potentiation of cellular cytotoxicity

**Fig. 3:**
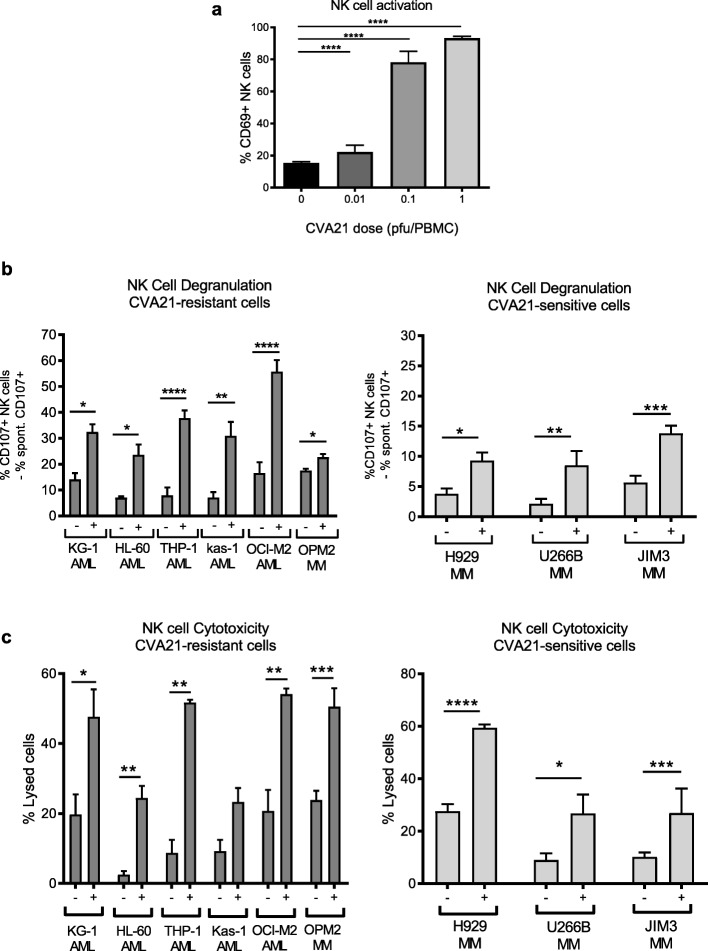
CVA21 treatment enhances NK cell activation and function. **a**. CD69 expression on healthy donor NK cells (CD3^−^CD56^+^) following CVA21 treatment for 48 h (*n* = 4). **b**. Healthy donor PBMC (± 0.1 pfu/cell CVA21) were co-cultured at a 2:1 ratio with AML or MM target cells for 5 h and the percentage of NK cells expressing CD107a/b was determined (*n* = 4); data for CVA21-sensitive (right) and CVA21-resistant (left) cell lines are shown. **c**. CVA21-sensitive (right) and CVA21-resistant (left) cell lines were labelled with ^51^Cr and then co-cultured with healthy donor PBMC (±CVA21 treatment) for 4 h and the percentage lysis of target cells was determined (*n* = 4). Error bars indicate SEM. *denotes statistical significance

We have previously shown that OV-induced type I IFN-α can increase the anti-tumor properties of NK cells [7], therefore given that CVA21 stimulated IFN-α in vitro (Fig. 2a), and activated NK cells in vivo (Fig. 1d), we examined the ability of OV-activated NK cells to eradicate CVA21-sensitive and CVA21-resistant cells. Initially we confirmed that CVA21 could activate NK cells, in vitro, and demonstrated that treatment of HD-PBMCs with CVA21 induced a significant increase in CD69 expression on NK cells, as expected (Fig. 3a). Pivotally, this heightened state of NK cell activation was associated with improved recognition and killing of both CVA21-sensitive (H929, U266B and JIM3) and CVA21-resistant (AML cell lines and OPM2) cell targets, as measured by CD107a/b expression (Fig. 3b) and chromium release (Fig. 3c), respectively. This confirmed the ability of CVA21 to induce NK cell mediated anti-tumor immunity.

### Priming of tumor-specific cytotoxic T cells using CVA21

**Fig. 4:**
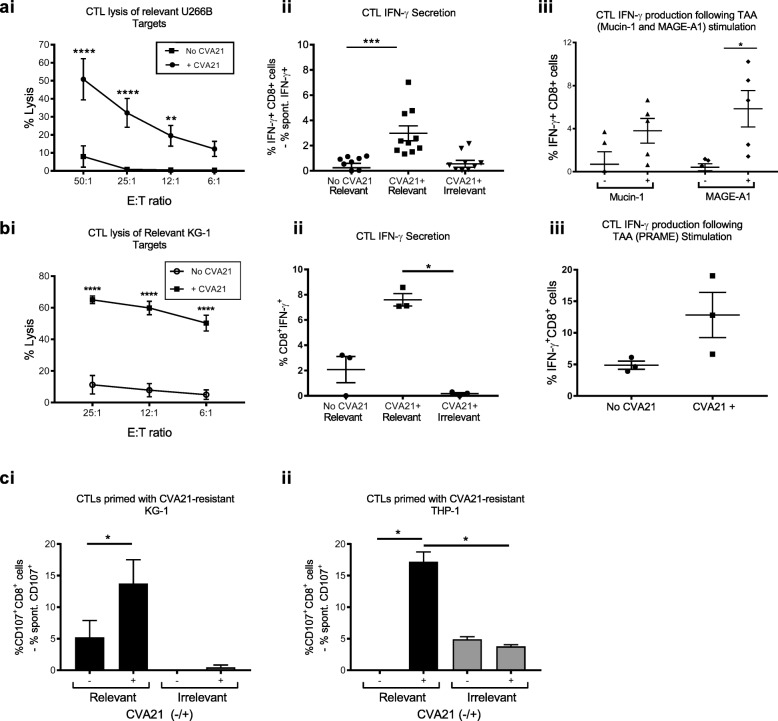
CVA21 can prime tumor-specific CTL. **a.** and **b**: CVA21-sensitive U266B MM (**a**) and ICAM-1/KG-1 (**b**) cell targets were used. Tumor cells were pre-treated with CVA21 (0.1 pfu/cell) for 24 h, then loaded onto mDC prior to being co-cultured with autologous PBMC and one round of re-stimulation. **a.** and **bi**. CTLs primed in the presence or absence of CVA21 were co-cultured with ^51^Cr-labelled relevant targets (U266B and ICAM-1/KG-1 cells, respectively) at different effector:target ratios for 4 h. The percent cell lysis was determined using ^51^Cr release (*n* = 6). **a.** and **bii**. CTL intracellular IFN-γ production following a 5 h co-culture with relevant (U266B or ICAM-1/KG-1, respectively) or irrelevant (ICAM-1/KG-1 or Raji, respectively) targets (*n* = 3). **a.** and **biii**. Intracellular IFN-γ production following a 5 h co-culture with autologous CD14^+^ cells loaded with appropriate peptide pools (Mucin-1 and MAGE-A1; U266B CTLs, and PRAME; ICAM-1/KG-1 primed CTLs)**. c.** CTL priming with CVA21-resistant cells (parental-KG-1 (**i**) and THP-1 (**ii**)). The percentage of tumor specific CTLs (CD3^+^CD8^+^) was determined using CD107a/b degranulation after a 5 h co-culture with relevant (KG-1; n = 3 or THP-1; *n* = 2) or irrelevant (Raji) cell targets. Spontaneous CD107 expression was subtracted from the values shown. Error bars indicate SEM. *denotes statistical significance

While innate immunity is rapid and instrumental for the eradication of tumor cells, generation of adaptive anti-tumor immunity is necessary for long-term immunological memory. The activation of both CD4^+^ and CD8^+^ T cells in cancer patients following CVA21 treatment (Fig. 1e and f) suggests the induction of a T cell immune response; however, its relationship to anti-viral and/or anti-tumor immunity is unclear. To evaluate the ability of CVA21 to stimulate the production of tumor-specific CTLs, we adapted our previously established protocol for OV CTL priming [8], to the hematological setting. This protocol involved long-term co-culture of CVA21-infected tumor cell targets, pre-loaded onto myeloid-derived dendritic cells (mDC), with PBMC autologous to the tumor-loaded mDC [8]. We initially examined the ability of CVA21 to stimulate CTL priming using CVA21-sensitive cells (U266B MM cells and ICAM-1/KG-1 AML cells) which, following direct oncolysis, should release damage-associated molecular patterns (DAMPs) and pathogen-associated molecular patterns (PAMPs) to activate mDC and facilitate CTL priming. Firstly, we identified that for efficient CTL priming, and lysis of relevant cell targets, the presence of CVA21 was required (Fig. 4Ai and Bi); moreover, primed-CTL were tumor specific, as only relevant, but not irrelevant, target cells were capable of stimulating intracellular IFN-γ production (Fig. 4Aii and Bii).

Next, to explore the antigen recognition repertoire of the primed-CTLs, we examined their responsiveness towards a known LAA (leukemia-associated antigen), Mucin-1 and Melanoma-associated antigen (MAGE)-A1 expressed by U266B cells [42], and PRAME (PReferentially expressed Antigen in Melanoma), expressed by KG-1 cell targets [43]. To do this, primed-CTLs were co-cultured with autologous CD14^+^ cells, pre-loaded with appropriate peptide pools, and intracellular IFN-γ production was quantified by flow cytometry. This readout allows tracking of T cell responses against known TAA/LAA without HLA restriction; autologous monocytes process and present peptides, which span the full length antigen, for stimulation of antigen-specific T cells and IFN-γ production. Although, as expected, inter-donor variation was observed, antigen-specific CTLs recognizing: 1) Mucin-1 and MAGE-A1, after priming with U266B cells, and 2) PRAME, after priming with ICAM-1/KG-1 cells, were identified (Fig. 4aiii and biii).

We have previously demonstrated, using an alternative OV, reovirus, that anti-tumour immunity can occur independently of direct oncolysis [44], therefore, to determine if CVA21-mediated oncolysis was required for the successful generation of CTLs, priming assays were repeated using parental-KG-1 and THP-1 cells which, by comparison to ICAM-1/KG-1 and U266B cells, were relatively non-permissive to CVA21 infection and oncolysis; for example, no evidence of viral replication or cell death was observed in THP-1 cells, and only low level replication (~ 130 fold increase in titre at 72 h) and cell death (~ 10% increase at 1pfu/cell) was observed in KG-1 (data not shown and Additional file 3: Figure S2B, respectively). Interestingly, these data demonstrate that tumor-specific CTLs were produced for both parental-KG-1 and THP-1 cell targets (Fig. 4C), indicating that CVA21-induced oncolysis (particularly for THP-1 cells) was not a pre-requisite for the generation of long-term anti-tumor immunity, as previously observed for reovirus [44].

### CVA21 maturation of mDC

**Fig. 5:**
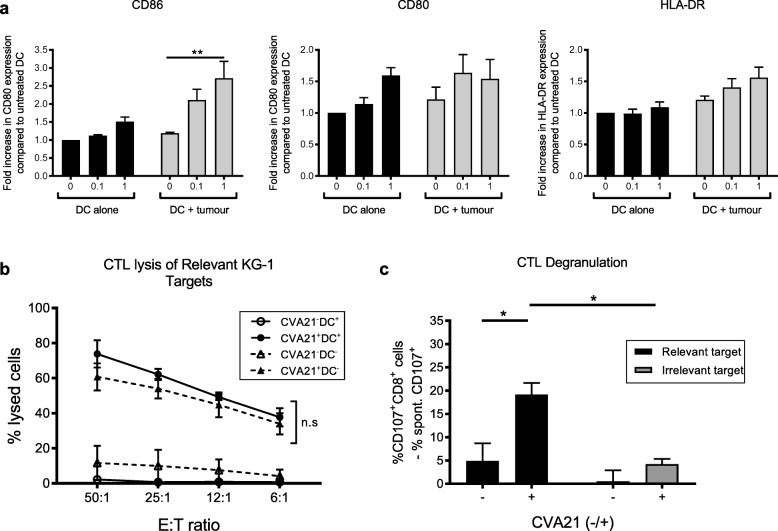
mDC are not necessary for priming of AML-specific CTL. **a**. mDC were treated with CVA21 or CVA21-treated ICAM-1/KG-1 cells for 48 h and expression of the activation markers, CD86, CD80 or HLA-DR, was examined (n = 4). **b**. ICAM-1/KG1-specific CTLs were primed with or without autologous mDC (±CVA21) and CTL-mediated lysis of relevant ICAM-1/KG-1 targets was measured by ^51^Cr release assay. Solid lines indicate CTL primed in the presence of mDC, dashed lines indicate CTL primed in the absence of mDC (*n* = 3). **c**. ICAM-1/KG1 CTLs, primed in the absence of mDC, were co-cultured with relevant (ICAM-1/KG-1) or irrelevant (Raji) target cells (n = 3) for 5 h and tumor specificity was examined using CD107a/b degranulation. Error bars indicate SEM. *denotes statistical significance

Given the efficient CTL priming demonstrated in Fig. 4, it was postulated that CVA21 would induce DC maturation following co-culture with CVA21-infected cell targets, and thus provide the necessary antigen presentation and co-stimulation to support efficient CTL priming. Surprisingly, upon phenotyping of mDC following treatment with CVA21 alone, or CVA21-loaded targets, limited mDC maturation was observed; CVA21 was unable to directly stimulate mDC maturation and only modest maturation (a small but significant increase in CD86 expression) was observed upon co-culture of mDC with CVA21-treated ICAM-1/KG-1 (Fig. 5a). Therefore, the importance for mDC during the course of the CTL priming assay was examined. To investigate this, we compared the ability of CVA21 to prime tumor-specific CTLs in the presence or absence of ex vivo generated, tumor-loaded, mDC. Figure 5b shows that CTLs primed with or without mDC were comparable in their capacity to lyse relevant tumor cell targets, demonstrating that CVA21-treated tumor cells can support CTL priming, irrespective of the presence or absence of mDC. Importantly, the CTLs generated in the absence of mDC retained their tumor specificity as CTL degranulation was only observed upon recognition of relevant, but not irrelevant, cell targets (Fig. 5c). Collectively, these data confirmed that in vitro generated mDC were not required for successful CTL priming, and suggested that all cellular components required for adaptive CTL priming, by CVA21-treated tumor cells, were present in the peripheral blood. This finding is of particular importance in the context of hematological malignances as CVA21-loaded tumor cells may co-exist, in the blood, with immune cell components that are necessary for effective CTL priming.

### ICAM-1 expression on immune cells is required for the induction of CVA21 anti-tumor immunity

**Fig. 6:**
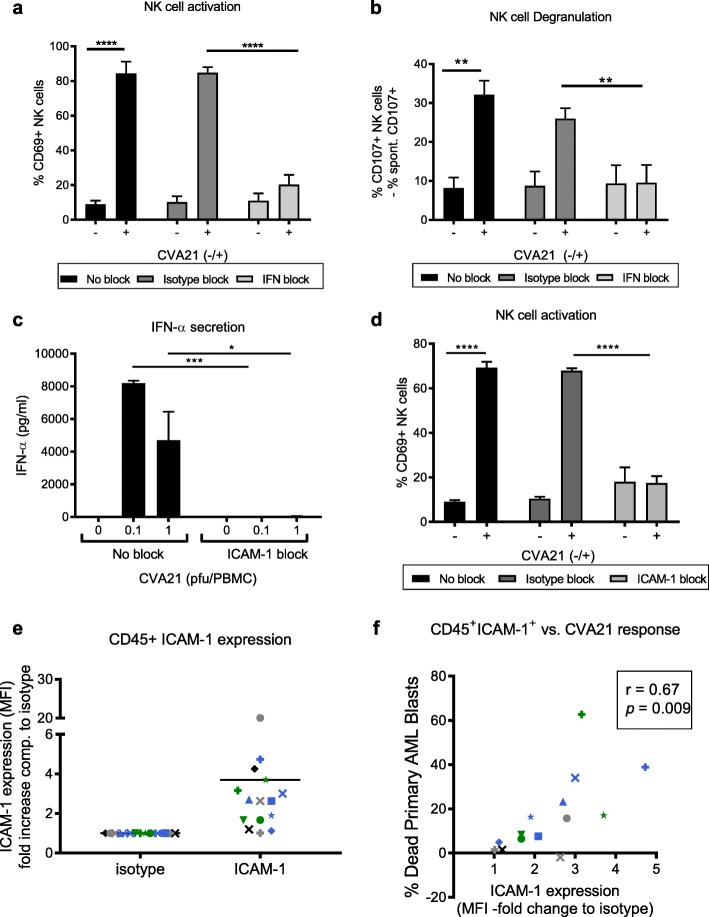
Type I IFN and ICAM-1 are required for CVA21-induced anti-tumor immunity. **a-d**. HD-PBMC were treated with CVA21 for 24 h, with or without pre-treatment with type-1 IFN blockade or an ICAM-1-blocking antibody. NK cell CD69 expression (**a**) and NK cell CD107a/b degranulation (**b**) were measured in the presence of type I IFN blocking antibodies or corresponding isotype antibodies (*n* = 4). **c**. HD-PBMC were treated with CVA21, in the presence or absence of ICAM-1-blocking antibodies, and IFN-α secretion was examined by ELISA (*n* = 3). **d**. NK cell CD69 expression was determined following CVA21treatment, with or without pre-treatment with an ICAM-1-blocking antibody or isotype control (*n* = 3). **e**. ICAM-1 expression was measured on mature hematopoietic immune cells (CD45^+^) from primary AML patients (*n* = 14). **f**. Correlation of CVA21 response (death of AML blasts) with ICAM-1 expression on mature CD45+ hematopoietic cells from primary AML samples. Error bars indicate SEM. *denotes statistical significance

Data presented in Figs. 1, 2, 3, 4 and 5 demonstrate that CVA21 can activate innate and adaptive anti-tumor immunity against tumor cells which are both sensitive and resistant to CVA21-direct oncolysis; moreover, it appeared that all the cellular components required for this to occur were present in the blood. Therefore, to identify potential biomarkers of CVA21 response we sought to further characterize the molecular and cellular determinants required for CVA21-mediated immune activation. Our preliminary studies suggested that CVA21 was unable to directly activate isolated NK cells (data not shown), therefore, in the context of PBMC, we initially examined the role of anti-viral type I IFN for the induction of CVA21-mediated NK cell anti-tumor immunity. Using monoclonal type I IFN-blocking antibodies, we confirmed that NK cell activation was mediated by type I IFNs, as NK cell CD69 upregulation (Fig. 6a) and increased NK cell degranulation (Fig. 6b), following CVA21 treatment of PBMC, was not observed when type I IFN signaling was inhibited. Moreover, following this the importance of ICAM-1 (required for CVA21 infection of tumor cells [24]) on immune cell components was investigated; blockade of ICAM-1 within PBMC, prior to and during CVA21 treatment, completely abrogated the secretion of IFN-α (Fig. 6c) and prevented NK cell activation (no increase in CD69 expression; Fig. 6d) demonstrating a significant role for ICAM-1 in mediating CVA21-induced immune activation.

To further elucidate the role for ICAM-1 (on tumor cells or immune effectors) in coordinating CVA21 efficacy, we took advantage of AML patient samples (see Additional file 5: Table S2). Ex vivo treatment of primary AML blasts with CVA21 identified a number of patients (*n* = 8 of 16) whose blasts were susceptible to CVA21 killing (see Additional file 6: Figure S4A), whilst CD45^+^ hematopoietic non-malignant cells remained unharmed (Additional file 6: Figure S4B). Initially, to explore whether CVA21 efficacy was dependent on direct oncolysis we examined the association between CVA21-induced death and ICAM-1 expression on malignant AML blasts; interestingly, no correlation was observed (Pearson’s r = 0.122, see Additional file 6: Figure S4C). Furthermore, no detectable viral replication was observed in nine out of the 10 samples tested, with only low-level viral replication observed in one sample (data not shown). Whilst disappointing in terms of the capacity of CVA21 to directly infect and lyse AML cells, these data were in accordance with our in vitro cell line data (see Additional file 3: Figure S2B and Additional file 4: Figure S3C) which suggested that AML cell lines were relatively non-permissive to CVA21-direct oncolysis.

Given the capacity of CVA21 to induce innate and adaptive anti-tumor immune responses (Figs. 1, 2, 3, 4 and 5), it was therefore postulated that the immunotherapeutic properties of CVA21 could be responsible for death of primary AML blasts. In support of this we have demonstrated that: patient-derived, CVA21-treated PBMC-CM was cytotoxic against AML cell lines (Additional file 6: Figure S4D); IFN-α, which stimulates immune cell activation but abrogates CVA21-direct oncolysis, was induced in CVA21 treated AML patient samples (Additional file 6: Figure S4E); patient NK cells were activated by CVA21 to increase CD69 expression (Additional file 6: Figure S4F); and that NK cell activation correlated with IFN-α production (Pearson’s r = 0.74, *p* = 0.0009; Additional file 6: Figure S4G). Furthermore, ICAM-1 expression on non-malignant CD45^+^ immune effector cells within patient samples (Fig. 6e) significantly correlated with the efficacy of CVA21 (death of patient AML blasts) in these primary patient samples (Pearson’s r = 0.67, *p* = 0.009; Fig. 6f). Therefore, within a mixed cell population, comprising patient AML blasts with autologous non-malignant CD45^+^ immune effector cells, the susceptibility of AML cells to CVA21 treatment was determined by ICAM-1 expression on immune effector cells, not the malignant AML compartment. Thus, whilst the exact immune mechanisms responsible for CVA21 cytotoxicity towards AML blasts have not been defined, these data support a role for CVA21-induced immunotherapy for CVA21 efficacy in AML patient samples, and demonstrated the importance of ICAM-1 in mediating this immune response.

### Plasmacytoid dendritic cells (pDC) are essential for induction of CVA21-mediated anti-tumor immunity

**Fig. 7:**
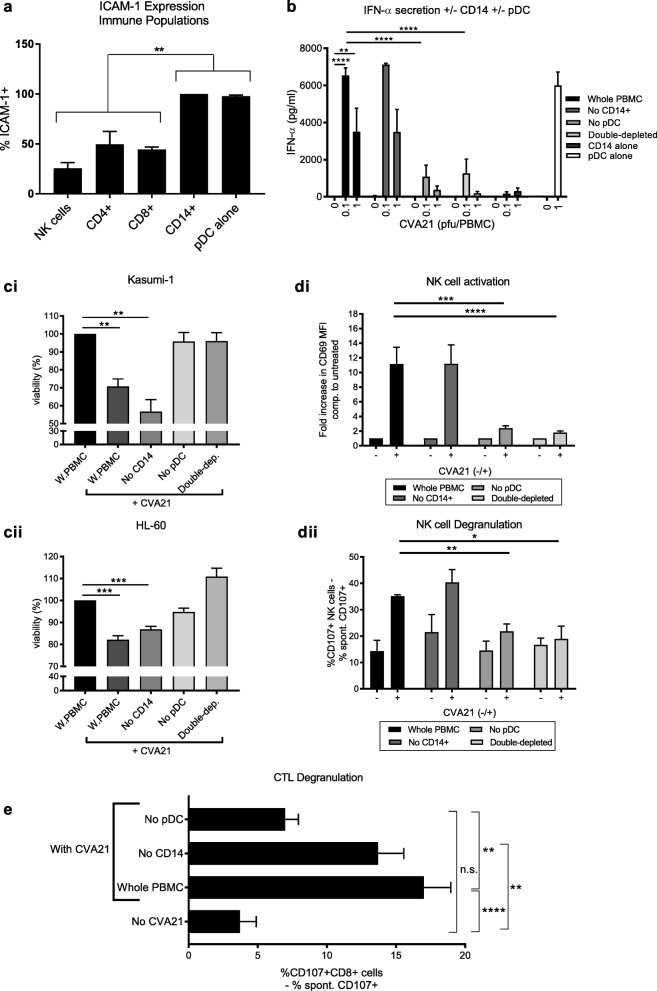
pDC orchestrate innate and adaptive CVA21 anti-tumor immunity. **a**. ICAM-1 expression on immune cell components from healthy donors (n = 3). **b-e** CD14^+^ monocytes and pDC (CD123^+^BDCA-2^+^) were depleted from whole PBMC (W.PBMC) prior to analysis. **b**. IFN-α secretion from whole or depleted PBMC, or isolated CD14^+^ cells and pDC, was measured by ELISA 48 h post-CVA21 treatment. **c**. CM was generated from whole or depleted PBMC, following treatment with 0.1 pfu/PBMC CVA21 for 48 h. The cytotoxicity of CM against kasumi-1 (**i**) and HL-60 (**ii**) cells after 96 h was evaluated by MTS assay. **d**. NK cell CD69 expression (**i**) and NK cell CD107a/b degranulation (**ii**) after treatment of whole of depleted PBMC with 0.1pfu/cell CVA21 for 48 h was determined. **e**. ICAM/KG-1 cells (±CVA21 and without addition of mDC) were used to prime CTL and PBMCs depleted of CD14^+^ monocytes or pDC were used as effector cells. Tumor-specific CTLs were detected using CD107a/b degranulation assays against cell targets. Error bars indicate SEM. *denotes statistical significance. n.s. = not significant

As ICAM-1 was identified as a key mediator of CVA21 anti-tumor immunity, we next sought to identify the immune cell component responsible for CVA21 recognition, and downstream immune activation. Initially, we identified both monocytes (CD14^+^) and pDC as cell populations which expressed significantly more ICAM-1 than NK cells, CD4^+^ and CD8^+^ T cells (Fig. 7A), therefore, we hypothesized that these two cell types may act as key regulators of CVA21-mediated anti-tumor immunity. To test this, we first examined IFN-α production (a key mediator of NK cell activation; Fig. 6) from PBMC depleted of CD14^+^ monocytes, pDC, or both (CD14^+^ cells and pDC), along with isolated CD14^+^ monocytes and pDC. Figure 7B demonstrates that pDC, in isolation, secreted large amounts of IFN-α following CVA21 treatment, and that IFN-α secretion from PBMC was abrogated following pDC depletion. By contrast, CD14^+^ depletion had no significant effect on IFN-α levels following CVA21 treatment, and isolated CD14^+^ cells did not secrete IFN-α in response to CVA21 treatment. To further examine the role of monocytes and pDC for anti-tumor immunity, we repeated PBMC-CM toxicity, NK cell activation and degranulation assays, as well as T cell priming experiments but depleted CD14^+^ monocytes, pDC, or both, from the whole PBMC population. Interestingly, CVA21-treated PBMC-CM remained toxic to kasumi-1 and HL-60 cells in the absence of CD14^+^ cells; however, when PBMC were depleted of pDC, the cytotoxicity of CVA21-treated PBMC-CM was reduced to levels comparable with untreated PBMC-CM (Fig. 7C). Moreover, the absence of IFN-α following pDC depletion (but not monocyte depletion), abrogated CVA21-induced NK cell activation with regards to both CD69 up-regulation, and enhanced NK cell degranulation (Fig. 7D). Furthermore, CTL priming assays (performed without the addition of autologous mDC) also revealed the importance of pDC, as the absence of CD14^+^ did not significantly decrease the production of tumor-specific CTLs; however, removal of pDC significantly reduced levels of tumor-specific CTL (Fig. 7E). Taken together, these results demonstrate, for the first time, the critical role of pDC in orchestrating CVA21-induced innate and adaptive anti-tumor immune responses and confirm the immunotherapeutic potential of this agent.

## Discussion

OVs represent a promising therapy for a wide range of solid malignancies, but still remain an under-investigated treatment option for hematological malignancies, despite easy intravenous access to both tumor and immune effector cells in these malignancies. The results presented here demonstrate the potential for efficient OVT against both AML and MM, despite AML cells being relatively resistant to CVA21-direct oncolysis. We have demonstrated that CVA21 can potentiate: 1) innate anti-tumor immunity, mediated both by cytokine-induced bystander killing and activation of NK cells; and 2) adaptive anti-tumor immunity against known TAA [45]. Mechanistically we have also demonstrated the importance of ICAM-1 on immune cell components, identified pDC as key orchestrators of CVA21-induced immunotherapy, and established that everything required to boost CVA21 immunity is available in the blood, which may be of particular significance in the hematological disease setting. Importantly, a role for CVA21-induced immunotherapy was also revealed for tumor cells which were both sensitive and resistant to CVA21-direct oncolysis, potentially widening the clinical applicability of this agent. Whilst we do not fully understand the mechanism/s by which CVA21 acts to boost CTL responses in less permissive tumor cells, it is possible that viral attachment to cell surface DAF (which is expressed by THP-1 and Kasumi-1; data not shown) or ICAM-1 which is expressed, albeit at low levels, facilitates immune activation and subsequent priming of CTLs. Access to blood samples from cancer patients taking part in the STORM (VLA009A) clinical trial (a Phase I dose escalation study of i.v. CVA21) enabled us to explore immune changes that occur in the blood following intravenous delivery of CVA21. These studies showed that interferon-stimulated genes (ISG) were induced 3 days after the first CVA21 infusion; moreover, at day 3 CD69 expression on NK cells, CD4+ T cells and CD8+ T cells was also increased (Fig. 1d-f) but decreased by day 22. Transient expression of CD69 on NK cells, associated with an IFN response, has previously been reported for reovirus, a dsRNA oncolytic virus [11]. Currently, the exact function of CD69 on lymphocytes remains unclear although CD69 has been implicated in both cell adhesion/migration and nutrient uptake. For example, CD69 expression can influence the migration and retention of lymphocytes within lymphoid tissue, and facilitate cell-to-cell interactions with APC via Gal-1. Additionally, CD69 contributes to the stability of LAT-1 (required for amino acid transport) on the plasma membrane of lymphocytes to facilitate nutrient uptake to sustain the activation and proliferation lymphocytes [46, 47]. The transient nature of CD69+ lymphocytes in the blood of patients after CVA21 treatment was not unexpected as CD69 is considered an early marker of lymphocyte activation which can be induced quickly but also declines rapidly following stimulation [46]. Unfortunately, as tumor biopsies were not available during this study it is unclear whether CD69+ lymphocytes persist in CVA21 treated patients, at sites other than the blood (i.e. lymph node and/or tumour), or whether CD69 was downregulated on lymphocytes as a mechanism of immune control once adaptive/humoral immunity had been appropriately triggered.

The dependence on pDC for detection and initiation of an immune response, resulting from abundant secretion of IFN-α, is in contrast to other OVs, namely reovirus, where monocytes were identified as key detectors in the peripheral blood [7], and could ultimately be used to inform patient stratification and predict responsiveness to CVA21 therapy. For example, personalized medicine approaches could be considered targeting patients with normal to high pDC levels, or patients with high expression of ICAM-1 on immune cell subsets and/or malignant blasts. Both of which could be easily assessed by flow cytometry on peripheral blood samples. Interestingly, pDC are increased in the bone marrow of MM patients, and whilst they are believed to contribute to immune cell dysfunction within the tumor microenvironment, engagement of pDC pattern recognition receptors (PRR), namely TLR-9, can restore pDC function and promote T cell proliferation [48] - a concept worth considering in the context of CVA21 engagement of alternative PRRs.

Whilst the direct lytic potential of CVA21 against primary AML was disappointing, the capacity of CVA21 to modulate anti-tumor immunity in the absence of lytic killing remains encouraging for patients with a more competent immune system, such as patients with MRD (minimal residual disease) or early relapse, when disease burden may be low; patients in remission have a reconstituted immune response with functional NK and T cells [49, 50]. Furthermore, to maximize OVT, the development of novel combination approaches should be prioritized. For example, combination with histone deacetylase inhibitors (HDACi), such as valproic acid (VPA), could be evaluated to: 1) increase the expression of NKG2D ligands on malignant blasts [51] and boost the cytotoxic effect of NK cells following OVT, or 2) synergise with OVs to increase viral replication and oncolysis [52]. In addition, hypomethylating agents, such as decitabine, can increase the expression of TAAs [45], therefore combination of CVA21 with different epigenetic modulators may be advantageous to boost both innate and adaptive anti-tumor immune mechanisms.

## Conclusion

In summary, we have shown that CVA21 can trigger anti-tumor responses against hematological malignancies. Moreover, pDC are central to detection of the virus in the circulation, and to subsequent priming of both the innate and adaptive arms of the immune response. For successful anti-TAA human CTL priming by CVA21-infected tumor cells, PBMCs suffice as a source of APC and responder T cells; therefore, in the context of hematological malignancies treated by systemic OVT, all the cellular components necessary for virus-mediated immunotherapy (including the tumor cells as an antigen source, as well as responder immune cells) are readily accessible. Overall, these data support the testing of intravenous CVA21 for the treatment of AML and MM, particularly in patients with a low disease burden and, potentially, in combination with other immunotherapies such as checkpoint inhibitors. Furthermore, the capacity of CVA21 to boost immunotherapeutic responses, despite relative resistance to CVA21-direct oncolysis, broadens the clinical applicability of this agent.

## Additional files

Additional file 1: **Table S1.** Details of flow cytometry antibodies used in the study. (DOCX 15 kb)

Additional file 2: **Figure S1.** CVA21 does not kill or replicate in healthy donor immune cells. (DOCX 183 kb)

Additional file 3: **Figure S2.** Susceptibility of AML and MM cell lines to CVA21 direct oncolysis. (DOCX 242 kb)

Additional file 4: **Figure S3.** Correlation of ICAM-1 expression and CVA21 susceptibility. (DOCX 470 kb)

Additional file 5: **Table S2.** Details of AML patients included in the study. (DOCX 16 kb)

Additional file 6: **Figure S4.** Efficacy of CVA21 against primary AML samples. A-B. (DOCX 796 kb)

## Data Availability

All data generated or analyzed during this study are included in this published article and supplementary material. Computational datasets were not generated or used in this study.

## Abbreviations

AML: Acute myeloid leukemia
APC: Antigen presenting cell
CTL: Cytotoxic T lymphocyte
CVA21: Coxsackievirus A21
DAF: Decay accelerating factor
DAMPs: Damage-associated molecular patterns
DC: Dendritic cells
HDAC: Histone deacetylase
HSV: Herpes simplex virus
ICAM-1: Intercellular adhesion molecule 1
mDC: Myeloid –derived DC
MM: Multiple myeloma
NK cell: Natural killer cells
OV: Oncolytic virus
OVT: Oncolytic virotherapy
PAMPs: Pathogen-associated molecular patterns
PBMC: Peripheral blood mononuclear cells
pDC: Plasmacytoid DC
TAA: Tumor-associated antigen
VPA: Valproic acid
VSV: Vesicular stomatitis virus
