# Supplementary material for: Plasmacytoid dendritic cells orchestrate innate and adaptive anti-tumor immunity induced by oncolytic coxsackievirus A21
Source: J Immunother Cancer. 2019 Jul 1;7:164. doi: 10.1186/s40425-019-0632-y (PMC6604201; doi:10.1186/s40425-019-0632-y)
Supplement: Supplementary file 1 — Table S1. Details of flow cytometry antibodies used in the study. (DOCX 15 kb) [file 40425_2019_632_MOESM1_ESM.docx]

| **Assay** | **Surface marker** | **Fluorophore conjugation** | **Antibody supplier** |
| --- | --- | --- | --- |
| **AML blast cell/CD45^+^ cells phenotyping** | CD34 | VioBlue | Miltenyi |
|  | CD45 | FITC | Miltenyi |
|  | CD117 | PE-Vio770 | Miltenyi |
|  | CD54, MICA/B, ULBP-1, ULBP-2/5/6, PVR, Nectin-2 | PE | BD Biosciences/R&D Systems |
|  | IgG_2a_/IgG_2b_ | PE | BD Biosciences |
| **NK cell activation** | CD56 | PE | Miltenyi |
|  | CD3 | PerCP | BD Biosciences |
|  | CD69 | FITC | BD Biosciences |
|  | IgG_1_ | FITC | BD Biosciences |
| **NK cell degranulation** | CD56 | PE | Miltenyi |
|  | CD3 | PerCP | BD Biosciences |
|  | CD107a | FITC | BD Biosciences |
|  | CD107b | FITC | BD Biosciences |
| **pDC ICAM-1 phenotyping** | BDCA-2 | VioBlue | Miltenyi |
|  | CD123 | FITC | BD Biosciences |
|  | CD54 | PE | BD Biosciences |
|  | IgG_2b_ | PE | BD Biosciences |
| **cDC phenotyping** | CD80 | PE | BD Biosciences |
|  | CD86 | FITC | BD Biosciences |
|  | HLA-DR | PerCP | BD Biosciences |
|  | IgG_1_ | PE | BD Biosciences |
|  | IgG_1_ | FITC | BD Biosciences |
|  | IgG_2_ | PerCP | BD Biosciences |

| **Assay** | **Surface marker** | **Fluorophore conjugation** | **Antibody supplier** |
| --- | --- | --- | --- |
| **NK ligand phenotyping** | CD54, MICA/B, Nectin-2 | PE | BD Biosciences |
|  | ULBP-1, ULBP-2/5/6, PVR, Nectin-2 | PE | R&D Systems |
|  | IgG2a | PE | BD Biosciences |
|  | IgG2b | PE | BD Biosciences |
| **CD8^+^ T cell degranulation** | CD3 | VioBlue | Miltenyi |
|  | CD56 | PE | Miltenyi |
|  | CD8 | PerCP | BD Biosciences |
|  | CD107a, CD107b | FITC | BD Biosciences |
| **IFN-γ expression in CD8^+^ T cells** | CD3 | VioBlue | Miltenyi |
|  | CD56 | PE | Miltenyi |
|  | CD8 | PerCP | BD Biosciences |
|  | IFN-γ | FITC | Miltenyi |
| **Immune cell phenotyping**  **(NK cells, CD4^+^/ CD8^+^ T cells,**  **B cells, monocytes and purified pDC)** | CD56 | PE | Miltenyi |
|  | CD3 | PerCP | BD Biosciences |
|  | CD4 | FITC | BD Biosciences |
|  | CD8 | VioBlue | Miltenyi |
|  | CD20 | VioBlue | Miltenyi |
|  | CD14 | PE | BD Biosciences |
|  | CD16 | FITC | BD Biosciences |
|  | CD123 | FITC | BD Biosciences |
|  | BDCA-2 | VioBlue | Miltenyi |

**Supplementary Table S1. Details of flow cytometry antibodies used in the study.**
