# Supplementary material for: Plasmacytoid dendritic cells orchestrate innate and adaptive anti-tumor immunity induced by oncolytic coxsackievirus A21
Source: J Immunother Cancer. 2019 Jul 1;7:164. doi: 10.1186/s40425-019-0632-y (PMC6604201; doi:10.1186/s40425-019-0632-y)
Supplement: Supplementary file 2 — Figure S1. CVA21 does not kill or replicate in healthy donor immune cells. (DOCX 183 kb) [file 40425_2019_632_MOESM2_ESM.docx]

**Supplementary Figure S1: CVA21 does not kill or replicate in healthy donor immune cells.** PBMC were isolated from the peripheral blood of healthy volunteers. In **A**-**D**, cell death was determined using a Live/Dead® stain and appropriate antibodies to identify each cell type. The mean percentage of cell death of resting and activated NK cells **(A)**, CD4^+^ T cells **(B)** and CD8^+^ T cells **(C)** after treatment with CVA21 for 120hrs is presented. For activation, PBMC were treated with 10 μg/mL phytohemagglutinin (PHA) for 36hrs before CVA21 treatment (n=3). **D.** Death of CD14^+^/CD16^+^ monocytes following CVA21 treatment for 120hrs (n=4). **E.** Cell-free supernatants were collected from PBMC at 0hrs and 120hrs after CVA21 treatment and CVA21 replication was assessed using a standard TCID_50_ assays on Mel-624 melanoma cells (n=4). All error bars indicate SEM (standard error of the mean).
