# Supplementary material for: Plasmacytoid dendritic cells orchestrate innate and adaptive anti-tumor immunity induced by oncolytic coxsackievirus A21
Source: J Immunother Cancer. 2019 Jul 1;7:164. doi: 10.1186/s40425-019-0632-y (PMC6604201; doi:10.1186/s40425-019-0632-y)
Supplement: Supplementary file 3 — Figure S2. Susceptibility of AML and MM cell lines to CVA21 direct oncolysis. (DOCX 242 kb) [file 40425_2019_632_MOESM3_ESM.docx]

**Supplementary Figure S2: Susceptibility of AML and MM cell lines to CVA21 direct oncolysis.** ICAM-1 expression was evaluated on six different AML cell lines (**A**) and four MM cell lines (**C**). The mean fold increase (MFI) in expression compared to isotype is presented (n=3). Cell viability of AML cell lines (**B**) and MM cell lines (**D**) was determined 72hrs post-CVA21 treatment (0.1 and 1 pfu/cell) using a Live/Dead® discrimination stain. The mean percentage of dead cells is presented (n=4 for AML; n=3 for MM). Error bars show SEM. *denotes statistical significance.
