# Supplementary material for: Plasmacytoid dendritic cells orchestrate innate and adaptive anti-tumor immunity induced by oncolytic coxsackievirus A21
Source: J Immunother Cancer. 2019 Jul 1;7:164. doi: 10.1186/s40425-019-0632-y (PMC6604201; doi:10.1186/s40425-019-0632-y)
Supplement: Supplementary file 4 — Figure S3. Correlation of ICAM-1 expression and CVA21 susceptibility. (DOCX 470 kb) [file 40425_2019_632_MOESM4_ESM.docx]

**Supplementary Figure S3: Correlation of ICAM-1 expression and CVA21 susceptibility. A.** KG-1 cells were treated with recombinant human IFN-α or IFN-γ for 96hrs and cell death was evaluated using Live/Dead^®^ (n=3). **B.** THP-1 and Kasumi-1 cells were treated with sub-toxic doses of TNF-α and ICAM-1 expression was determined (n=3). **C.** THP-1 and Kasumi-1 cells were treated with TNF-α for 24 hrs and CVA21 (0.1 or 1 pfu/cell) for a further 72 hrs. Cell viability was determined using Live/Dead (n-=3). **D.** KG-1 cells were treated with TNF-α and ICAM-1 expression was determined (n=3). **E.** KG-1 cells were treated with TNF-α for 24 hrs and CVA21 (0.1 or 1 pfu/cell) for a further 72 hrs. Cell viability was determined using Live/Dead (n-=3). **F.** ICAM-1-transduced KG-1 cells (ICAM-1/KG-1) were treated with 1 pfu/cell CVA21 in the presence or absence of IFN-α concentrations for 72 hrs and cell viability was determined by Live/Dead (n=3). Error bars show SEM. *denotes statistical significance.
