# Supplementary material for: Plasmacytoid dendritic cells orchestrate innate and adaptive anti-tumor immunity induced by oncolytic coxsackievirus A21
Source: J Immunother Cancer. 2019 Jul 1;7:164. doi: 10.1186/s40425-019-0632-y (PMC6604201; doi:10.1186/s40425-019-0632-y)
Supplement: Supplementary file 5 — Table S2. Details of AML patients included in the study. (DOCX 16 kb) [file 40425_2019_632_MOESM5_ESM.docx]

| **Sample** | **Symbol** | **Age** | **Sex** | **AML subtype (WHO)** | **Known mutations** | **Cytogenetics** | |
| --- | --- | --- | --- | --- | --- | --- | --- |
| ***AML-1*** | • | 37 | F | NOS | None detected | | 5q del. |
| ***AML-2*** | ◼ | 30 | M | inv(16)(p13;q22) | *CFS3R, WT-1, c-Kit* | | 16 inv. |
| ***AML-3*** | ▼ | 74 | M | RAEB | *STAG2* | | Normal |
| ***AML-4*** | ▲ | 74 | F | NOS | Not done | | Not done |
| ***AML-5*** | ◆ | 43 | F | NOS | *CFS3R* | | 16q del? |
| ***AML-6*** | ★ | 78 | F | MDS → AML | Not done | | Not done |
| ***AML-7*** | ✚ | 61 | F | AML relapse (monoblastic) | *FLT3-ITD* | | Trisomy 8, Trisomy 11 |
| ***AML-8*** | **✕** | 72 | M | t(8;21)(q22;q22) | *FLT3-ITD, RUNX1-RUNX1T1* fusion | | t(8;21)(q22;q22) |
| ***AML-9*** | • | 88 | M | NOS | *FLT3-ITD, NPM1, DNMT3A* | | Normal |
| ***AML-10*** | ◼ | 70 | F | NOS | *DNMT3A, IDH2* | | Normal |
| ***AML-11*** | ▼ | 61 | M | NOS | *SRSF2, IDH1, TET2* | | Trisomy 8 |
| ***AML-12*** | ▲ | 60 | M | NOS | *NRAS, TET2* | | t(2;3), -7 |
| ***AML-13*** | ◆ | 58 | M | NOS | IDH2, NPM1 | | Normal |
| ***AML-14*** | ★ | 75 | M | AML with adverse cellular features | *FLT3-ITD, DNMT3A* | | Normal |
| ***AML-15*** | ✚ | 61 | F | t(8;21)(q22;q22) | *RUNX1-RUNX1T1* fusion | | t(8;21) |
| ***AML-16*** | ✕ | 35 | M | inv(16)(p13;q22) | *FLT3-TKD, c-Kit, TET2, EZH2* | | 16 inv. |
| ***AML-17*** | • | 68 | M | t(8;21)(q22;q22) | *RUNX1-RUNX1T1* fusion, *WT-1* | | t(8;21) |
| ***AML-18*** | ◼ | 47 | F | AML with MLL (KMT2A) (11q23) rearrangement | *WT-1* | | t(9;11)(p21.3;q23.3) |
| ***AML-19*** | ▼ | 53 | F | NOS | *NPM1, DNMT3A* | | Normal |
| ***AML-20*** | ▲ | 47 | F | inv(16)(p13;q22) | *CBFB-MYH11* fusion | | 16 inv. |
| ***AML-21*** | ◆ | 73 | F | t(8;21)(q22;q22) | *FLT3-ITD, RUNX1-RUNX1T1* fusion | | t(8;21)(q22;q22) |
| ***AML-22*** | ★ | 73 | M | MDS with excess blasts | None detected | | Normal |
| ***AML-23*** | ✚ | 55 | M | NOS | Not done | | Incomplete |
| ***AML-24*** | ✕ | 69 | M | AML:lymphoma mixed | None detected | | Complex |
| ***AML-25*** | • | 71 | F | inv(16)(p13;q22) | *CBFB-MYH11* fusion | | 16 inv. |
| ***AML-26*** | ◼ | 58 | M | NOS | *CSF3R, TP53* | | Complex |
| ***AML-27*** | ▼ | 70 | M | AML with mutated NPM1 | *NPM1, TET2, FLT3-ITD* | | Normal |
| ***AML-28*** | ▲ | 66 | M | NOS | *CSF3R, DNMT3A* | | Normal |
| ***AML-29*** | ◆ | 61 | M | AML with mutated NPM1 | *NPM1, TET2* | | Normal |
| ***AML-30*** | ★ | 67 | M | AML with mutated NPM1 | *NPM1, TET2* | | Normal |
| ***AML-31*** | ✚ | 29 | M | inv(16)(p13;q22) | *CBFB-MYH11* fusion | | 16 inv. |

**Supplementary Table S2. Details of AML patients included in the study.**
