# Supplementary material for: Plasmacytoid dendritic cells orchestrate innate and adaptive anti-tumor immunity induced by oncolytic coxsackievirus A21
Source: J Immunother Cancer. 2019 Jul 1;7:164. doi: 10.1186/s40425-019-0632-y (PMC6604201; doi:10.1186/s40425-019-0632-y)
Supplement: Supplementary file 6 — Figure S4. Efficacy of CVA21 against primary AML samples. A-B. (DOCX 796 kb) [file 40425_2019_632_MOESM6_ESM.docx]

**Supplementary Figure S4: Efficacy of CVA21 against primary AML samples. A-B.** Viability of AML blast cells (**A**) and non-malignant hematopoietic CD45^+^ immune cells (**B**) was evaluated six days post CVA21 treatment (n=16). Solid lines indicate responsive samples, dashed lines indicate non-responsive samples, defined using a >10% induction of cell death threshold. **C.** ICAM-1 expression on primary AML blasts (CD45^-^CD34^+^ and/or CD117^+^) at isolation was correlated with overall AML susceptibility to CVA21 6 days post-infection. **D.** KG-1, HL-60 and kasumi-1 cells were cultured for 96hrs in CM generated from primary AML patient PBMC (n=13 for KG-1, n=20 for HL-60, n=10 for kasumi-1) and cytotoxicity was determined using MTS assays. **E.** Primary AML samples were treated with CVA21 for 48hrs and IFN-α secretion was measured by ELISA (n=21). **F.** NK cell CD69 expression (n=23) was measured in primary samples from AML patients. Solid lines highlight samples where NK cell activation was observed (responders) and dashed lines indicate samples where no NK cells activation was observed (non-responders). **G.** Correlation of patient NK cell degranulation with IFN-α secretion following CVA21 treatment (n=16). *denotes statistical significance.
